# Short-term effects of ambient temperature on acute exacerbation of inflammatory bowel disease: A nationwide case-crossover study with external validation

**DOI:** 10.1371/journal.pone.0291713

**Published:** 2023-12-29

**Authors:** Yeong Chan Lee, Tae Jun Kim, Jong-Hun Kim, Eunjin Lee, Woong-Yang Park, Kyunga Kim, Hee Jung Son

**Affiliations:** 1 Sungkyunkwan University School of Medicine, Seoul, Republic of Korea; 2 Department of Digital Health, Samsung Advanced Institute for Health Sciences & Technology (SAIHST), Sungkyunkwan University, Samsung Medical Center, Seoul, Republic of Korea; 3 Department of Medicine, Samsung Medical Center, Sungkyunkwan University School of Medicine, Seoul, Republic of Korea; 4 Department of Social and Preventive Medicine, Sungkyunkwan University School of Medicine, Suwon, Republic of Korea; 5 Samsung Genome Institute, Samsung Medical Center, Seoul, Republic of Korea; 6 Biomedical Statistics Center, Research Institute for Future Medicine, Samsung Medical Center, Seoul, Republic of Korea; 7 Center for Health Promotion, Samsung Medical Center, Sungkyunkwan University School of Medicine, Seoul, Korea; Kyung Hee University School of Medicine, REPUBLIC OF KOREA

## Abstract

Inflammatory bowel disease (IBD) is an idiopathic inflammatory disorder characterized by chronic and relapsing manifestations. Several environmental factors are known as triggers for exacerbation of IBD. However, an association between exacerbation of IBD and ambient temperature is uncertain. This study aimed to estimate the risk of acute exacerbation of IBD due to ambient temperature. We performed a bidirectional case-crossover study using a nationwide claim data from South Korea. The external validation was conducted with a large prospective cohort in the United Kingdom. We confirmed significant associations between acute exacerbation of IBD and the short-term ambient temperature changes toward severe temperatures, in the cold weather (-19.4°C–4.3°C) (odd ratio [OR] = 1.13, 95% confidence interval [CI]: 1.13–1.14) and in the hot weather (21.3°C–33.5°C) (OR = 1.16, 95% CI: 1.15–1.17). However, the association was not significant in the moderate weather (4.3°C–21.3°C). The external validation suggested consistent results with additional elevation of acute exacerbation risk in the colder weather (-13.4°C to 2.6°C) (OR = 1.90, 95% CI: 1.62–2.22) and in the hotter weather (15.7°C–28.4°C) (OR = 1.41, 95% CI: 1.32–1.51). We observed and validated that the short-term ambient temperature changes were associated with acute exacerbation of IBD in the cold and hot weathers. Our findings provide evidence that temperature changes are associated with the acute exacerbation of IBD.

## Introduction

Inflammatory bowel disease (IBD) is a chronic inflammation that occurs in the gastrointestinal tract. Crohn’s disease (CD) and ulcerative colitis (UC) are the main types of IBD. The prevalence of IBD is increasing globally, besides life-years lived with the disability are still increasing [1]. The prevalence of IBD in South Korea has increased almost double during eight years from 2009 to 2016 [2]. The prevalence of IBD in the United Kingdom has still increased from 2006 to 2016 [3]. Because the disease pathway caused by the exogenous factors is uncertain, it is crucial to reveal unknown risk factors in epidemiological studies. Moreover, since IBD is a chronic disease, it is important to improve patients’ quality of life by reducing the risk of acute exacerbation.

IBD is considered to arise from the regulation of immune responses, the microbiome, and genetic or environmental components [4, 5]. Environmental factors such as living conditions or vitamin D deficiency may be associated with IBD according to epidemiologic evidence [5]. As IBD is associated with complex etiologies, ambient temperature, which affects the human body consistently and directly, is considered to be related to acute exacerbation of IBD. We conducted a meta-analysis and systematic review in our previous research and drew conclusions from it [6]. We confirmed a statistically significant relationship between acute exacerbation of IBD and seasonal variation. However, we noted inconsistencies across studies with regard to the specific season associated with IBD exacerbation. For example, one study found no significant association between cold weather spells and the exacerbation of IBD [7]. In contrast, a different study identified a significant correlation between heat waves and IBD exacerbation [8]. There was a seasonal trend of increasing remission due to acute exacerbation of IBD in summer in a pediatric study [9]. Meanwhile, a study on acute exacerbation of IBD at six hospitals in South Korea showed that CD occurred statistically higher in spring than in other seasons, but there was no seasonal variation for UC [10].

The prevalence of IBD in South Korea is still growing, but the association between ambient temperature and acute exacerbation of IBD has rarely been studied. We analyzed patients with IBD extracted from nationwide claims data covering almost the entire population in South Korea to investigate the association. We further considered a large prospective cohort from the UK to validate our hypothesis that changes in ambient temperature are associated with acute exacerbation of IBD.

## Materials and methods

### Data sources

We used the National Health Insurance Service (NHIS) claims database and meteorological and atmospheric quality data from the Korea Meteorological Administration (KMA) to investigate the association between short-term temperature changes and the risk of acute exacerbation of IBD. The NHIS provides researchers a customized population-based data from its claims database that includes approximately 50 million people, i.e., almost the entire population in South Korea. The database contains de-identified demographic information including sex, age, simplified residence up to district unit, as well as medical information including diagnosis history, drug prescription history, and results of health examinations. The study population was followed for 9 years, from 2009 to 2017.

Weather and air pollutant data collected by the KMA were available to the public. Hourly ambient temperature, relative humidity, coarse particulate matter (PM_10_), nitrogen dioxide (NO_2_), sulfur dioxide (SO_2_), carbon monoxide (CO), and ozone (O_3_) were measured at monitoring sites in South Korea. Daily average values of each variable were used in the analyses. Because the NHIS did not release the detailed residential information under the district for privacy purposes, claim data for each individual were matched with weather and air pollutant data from the closest monitoring site using the Haversine distance.

We externally validated our hypothesis using a large prospective cohort from the UK Biobank. The UK Biobank consists of approximately 500,000 healthy participants’ medical information, including phenotypes, restricted residential information, and medical history provided by national health registries [11]. The 40–69 middle-aged participants who resided in Scotland, England, and Wales were recruited between 2006 and 2010. They were followed up prospectively. The hospital inpatient records included diagnosis history and admission information.

We obtained the UK’s daily air temperature data from HadUK-Grid provided by Met Office, which comprises gridded climate variables [12]. The daily average temperature was calculated as the average of the gridded daily maximum and minimum temperatures converted with a 5-km resolution between 2011 and 2017. However, we couldn’t access the daily data for relative humidity, PM_10_, NO_2_, SO_2_, CO, and O_3_.

The study protocol was approved by the Institutional Review Board of the Samsung Medical Center (no.: 2020-03-162). This research was externally validated using the UK Biobank Resource under application no. 33002. Informed consent from participants of NHIS was not obtained because all data of NHIS were provided to researchers retrospectively with anonymization. All participants in the UK Biobank provided their written informed consent.

### Study design

**Fig 1:**
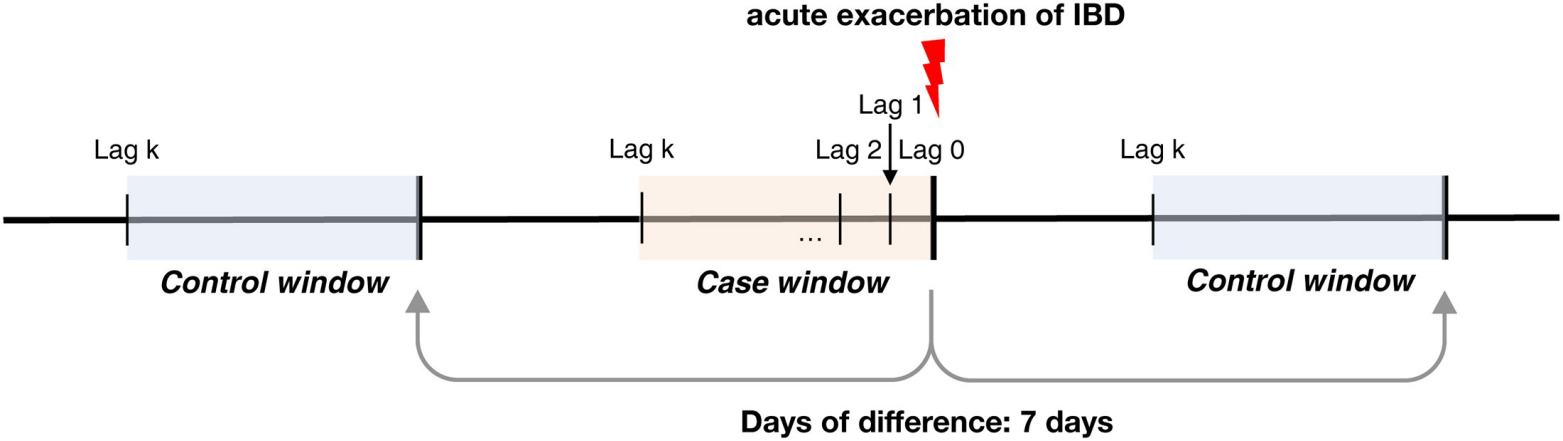
Illustration of bidirectional case-crossover design.

We used a bidirectional case-crossover design that is popular for studying the effects of short-term exposures for the risk of acute disease [13]. In bidirectional case-crossover design, control periods are selected both before and after the event. It thereby reduces bias that can occur if there are temporal trends in exposure or occurrence of the event. Each patient with a disease was compared to him/herself between different time periods, namely disease (case) versus non-disease (control) periods so that time-invariant factors, such as sex, or genetic factors, can be self-controlled. The case day for each individual was defined as the initial date of acute exacerbation of IBD, and two matched control days were defined on the same day of the week before or after the case day (Fig 1). This design can reduce the bias caused by directionality between the control and case period [14]. We defined single lags as time lags of from 1 to 6 days before the case and matched control days to explore possible lagged effects of short-term exposures. The cumulative effects of short-term exposures were also examined by using moving averages during lagged time intervals up to 6 days (lag 0–1 to lag 0–6).

### Study population

Among approximately 50 million individuals in the NHIS population-based cohort, 215,329 patients had been diagnosed with IBD (International Classification of Diseases version 10 [ICD-10] codes: either K50 as CD or K51 as UC) between January 1, 2009 and December 31, 2017. We selected 39,978 cases with acute exacerbation of IBD. The day of the occurrence of the acute exacerbation of IBD was considered to be the same as the day of the patient’s visit to the hospital and the diagnosis of the disease. The cases were identified as IBD patients who were prescribed steroids to treat the acute exacerbation. We have excluded considerations pertaining to biologics. Generally, the initiation of biologics such as TNF inhibitors is associated with step-up treatment in cases of ‘loss of response’ to existing treatments, situations that are not classified as acute exacerbations. There were 80,570 acute exacerbation episodes because some cases experienced multiple episodes. For each individual, a case day was defined as the occurrence date of the first episode, and a case period was defined as the period within 90 days after the case day. If another episode occurred within the case period, it was considered as a recurrent event of the initial acute exacerbation. Then the case period was extended by an additional 90 days from the date of the recurrent acute exacerbation. However, if another episode occurred outside the case period, it was considered as a new initial event and induced a new case period.

In the validation cohort from the UK Biobank, first of all, we identified 4,680 patients who had been diagnosed with IBD (ICD-10: K50 as CD, K51 as UC and ICD-9: 555 as CD, 556 as UC). Then, we selected 2,548 episodes of acute exacerbation of IBD from 1,493 patients who had been diagnosed with IBD at least once before January 1, 2011 and were admitted due to IBD. Because there was no prescription information for IBD, the acute exacerbation was defined as the admission to an inpatient facility due to IBD. The case days and periods were identified in the same manner.

### Statistical analysis

Conditional logistic regression was employed to investigate the short-term effects of the ambient temperature on the acute exacerbation of IBD. Crude odds ratios (ORs) were estimated in univariable analyses, and adjusted ORs were obtained by adding the daily average relative humidity, PM_10_, NO_2_, SO_2_, O_3_, and CO in the multivariable analyses [15]. To reduce bias due to seasonal variations in temperature, we divided the distribution of daily average temperatures during the observational periods into quartiles: cold, cool, warm and hot temperatures. The effects of temperature on the risk of acute exacerbation were analyzed in each quartile. The patients were further subdivided into CD and UC groups, and sensitivity analyses were conducted to investigate the potential impact in the subgroups. Statistical significance was declared when the two-sided *p*-value was < 0.05. Data processing and analyses were performed using SAS Enterprise Guide version 7.2 (SAS Institute Inc., Cary, NC, USA) and R software version 3.6.3 (R Foundation for Statistical Computing, Vienna, Austria).

## Results

In the NHIS database, 39,978 patients with acute exacerbation of IBD were included in this study. Among them, 14,118 patients (35.3%) were diagnosed with CD, and the other 25,860 patients (64.7%) were diagnosed with UC. Age of the patients is 40.2 ± 18.1 when the first acute exacerbation of IBD was aggravated. Table 1 shows the descriptive statistics of the study population (for the definitions see S1 Table). In the UK Biobank, of 1,493 patients with acute exacerbation of IBD, 508 and 985 patients were diagnosed with CD and UC, respectively.

**Table 1:** Characteristics of study participants.

	NHIS	UK Biobank
**No. patients**	39,978	1,493
CD	14,118 (35.3%)	508 (34.0%)
UC	25,860 (64.7%)	985 (66.0%)
**Age at the first acute exacerbation of IBD**	40.2 ± 18.1	61.3 ± 8.2
**Sex**		
Male	24,434 (61.1%)	735 (49.2%)
Female	15,544 (38.9%)	758 (50.8%)
**Comorbidities**		
Myocardial infarction	462 (1.2%)	109 (7.3%)
Congestive heart failure	1,575 (3.9%)	72 (4.8%)
Peripheral vascular disease	4,692 (11.7%)	91 (6.1%)
Cerebrovascular disease	3,694 (9.2%)	82 (5.5%)
Dementia	1,412 (3.5%)	29 (1.9%)
Chronic pulmonary disease	21,645 (54.1%)	344 (23.0%)
Connective tissue disease	3,570 (8.9%)	96 (6.4%)
Peptic ulcer disease	21,184 (53.0%)	90 (6.0%)
Diabetes (without complications)	5,513 (13.8%)	112 (7.5%)
Diabetes (with complications)	2,022 (5.1%)	207 (13.9%)
Paraplegia/hemiplegia	503 (1.3%)	17 (1.1%)
Mild liver disease	13,254 (33.2%)	11 (0.7%)
Moderate or severe liver disease	273 (0.7%)	28 (1.9%)
Renal disease	693 (1.7%)	135 (9.0%)
Malignancy	2,723 (6.8%)	363 (24.3%)
AIDS	40 (0.1%)	0 (0.0%)

* Abbreviation: NHIS, National Health Insurance Service; CD, Crohn’s diseases; UC, ulcerative colitis; IBD, inflammatory bowel disease; AIDS, acquired immunodeficiency syndrome.

Table 2 shows summary statistics of the daily average temperature, daily average relative humidity, and air pollutants for 80,570 episodes at lag 0 by total and quartile temperatures in the NHIS database, and the daily average temperature for 2,548 episodes in the UK Biobank. In the first quartile, the daily average temperature (mean±standard deviation; -0.74±3.62) measured on the date of acute exacerbation of IBD (lag 0) was significantly different compared with that (0.71±5.05) measured on the matched control days. In the fourth quartile, the daily average temperatures were significantly different between on the case days (24.90±2.31) and on the matched control days (24.36±2.98). This tendency was also observed in the other quartiles. The results of the daily average temperature in the quartiles in the UK Biobank had a similar pattern with those in the NHIS database. The daily average temperature in case periods was significantly lower than that in control periods in the first quartile (*p*<0.001), whereas it was significantly higher in fourth quartile (*p*<0.001).

**Table 2:** Summary statistics of episodes with a lag 0.

**KMA**	**Temperature quartile (°C)**	Total (-19.4–33.5)	P-value	Q1 (-19.4–4.3)	P-value	Q2 (4.3–13.7)	P-value	Q3 (13.7–21.3)	P-value	Q4 (21.3–33.5)	P-value
**NHIS**	**Acute exacerbation of IBD episodes (n, %)**	80,570 (100%)		19,509 (24.2%)		18,958 (23.5%)		18,486 (22.9%)		23,627 (29.3%)	
	**Daily average temperature (°C)**		0.981		<0.001		<0.001		<0.001		<0.001
	Case periods (mean ± S.D.)	13.29 ± 10.21		-0.74 ± 3.62		8.89 ± 2.83		17.78 ± 2.26		24.90 ± 2.31	
	Control periods (mean ± S.D.)	13.29 ± 10.24		0.71 ± 5.05		8.24 ± 5.47		17.59 ± 4.41		24.36 ± 2.98	
	**Daily average relative humidity (%)**		0.578		<0.001		<0.001		<0.001		<0.001
	Case periods (mean ± S.D.)	65.61 ± 16.29		56.75 ± 15.40		62.87 ± 16.20		67.33 ± 15.21		73.78 ± 13.36	
	Control periods (mean ± S.D.)	65.57 ± 13.31		59.41± 15.73		60.38 ± 16.57		65.68 ± 15.09		74.73 ± 12.98	
	**Air pollutants**										
	PM10 (10 *μg/m*^3^)		0.010		<0.001		<0.001		0.002		<0.001
	Case periods (mean ± S.D.)	49.65 ± 28.94		53.76 ± 31.14		57.29 ± 31.02		50.51 ± 30.04		39.25 ± 20.04	
	Control periods (mean ± S.D.)	49.33 ± 28.80		56.41 ± 31.83		55.49 ± 32.54		49.69 ± 26.78		38.01 ± 19.31	
	NO2 (10 ppb)		<0.001		<0.001		<0.001		<0.001		<0.001
	Case periods (mean ± S.D.)	27.00 ± 14.76		30.66 ± 15.34		30.43 ± 15.18		26.77 ± 14.58		21.33 ± 12.03	
	Control periods (mean ± S.D.)	26.73 ± 14.71		32.08 ± 15.67		28.95 ± 14.97		26.12 ± 14.11		20.96 ± 11.74	
	SO2 (1 ppb)		0.022		0.002		<0.001		0.007		0.012
	Case periods (mean ± S.D.)	5.09 ± 2.79		6.28 ± 3.07		5.38 ± 2.77		4.69 ± 2.58		4.17 ± 2.30	
	Control periods (mean ± S.D.)	5.06 ± 2.78		6.37 ± 3.13		5.29 ± 2.71		4.63 ± 2.43		4.12 ± 2.27	
	O3 (10 ppb)		0.039		<0.001		<0.001		<0.001		<0.001
	Case periods (mean ± S.D.)	24.10 ± 13.08		16.19 ± 9.10		21.82 ± 11.82		28.74 ± 13.02		28.87 ± 13.22	
	Control periods (mean ± S.D.)	24.22 ± 13.03		15.71 ± 9.08		22.88 ± 11.73		29.25 ± 13.34		28.4 ± 12.79	
	CO (1 ppm)		0.007		<0.001		<0.001		<0.001		0.070
	Case periods (mean ± S.D.)	0.54 ± 0.26		0.67 ± 0.31		0.59 ± 0.26		0.48 ± 0.19		0.42 ± 0.17	
	Control periods (mean ± S.D.)	0.53 ± 0.25		0.69 ± 0.31		0.56 ± 0.24		0.47 ± 0.18		0.42 ± 0.17	
**HadUK-Grid**	**Temperature quartile (°C)**	Total (-13.4–28.4)	P-value	Q1 (-13.4–5.5)	P-value	Q2 (5.5–9.2)	P-value	Q3 (9.2–13.0)	P-value	Q4 (13.0–28.4)	P-value
**UK Biobank**	**Acute exacerbation of IBD episodes (n, %)**	2,548 (100%)		484 (19.0%)		639 (25.1%)		596 (23.4%)		829 (32.5%)	
	**Daily average temperature (°C)**		0.891		<0.001		0.957		0.001		<0.001
	Case periods (mean ± S.D.)	13.25 ± 5.07		3.19 ± 1.80		7.31 ± 1.09		11.04 ± 1.06		16.08 ± 2.29	
	Control periods (mean ± S.D.)	13.24 ± 5.07		5.16 ± 3.07		7.30 ± 3.35		10.68 ± 3.51		15.15 ± 3.18	

**Fig 2:**
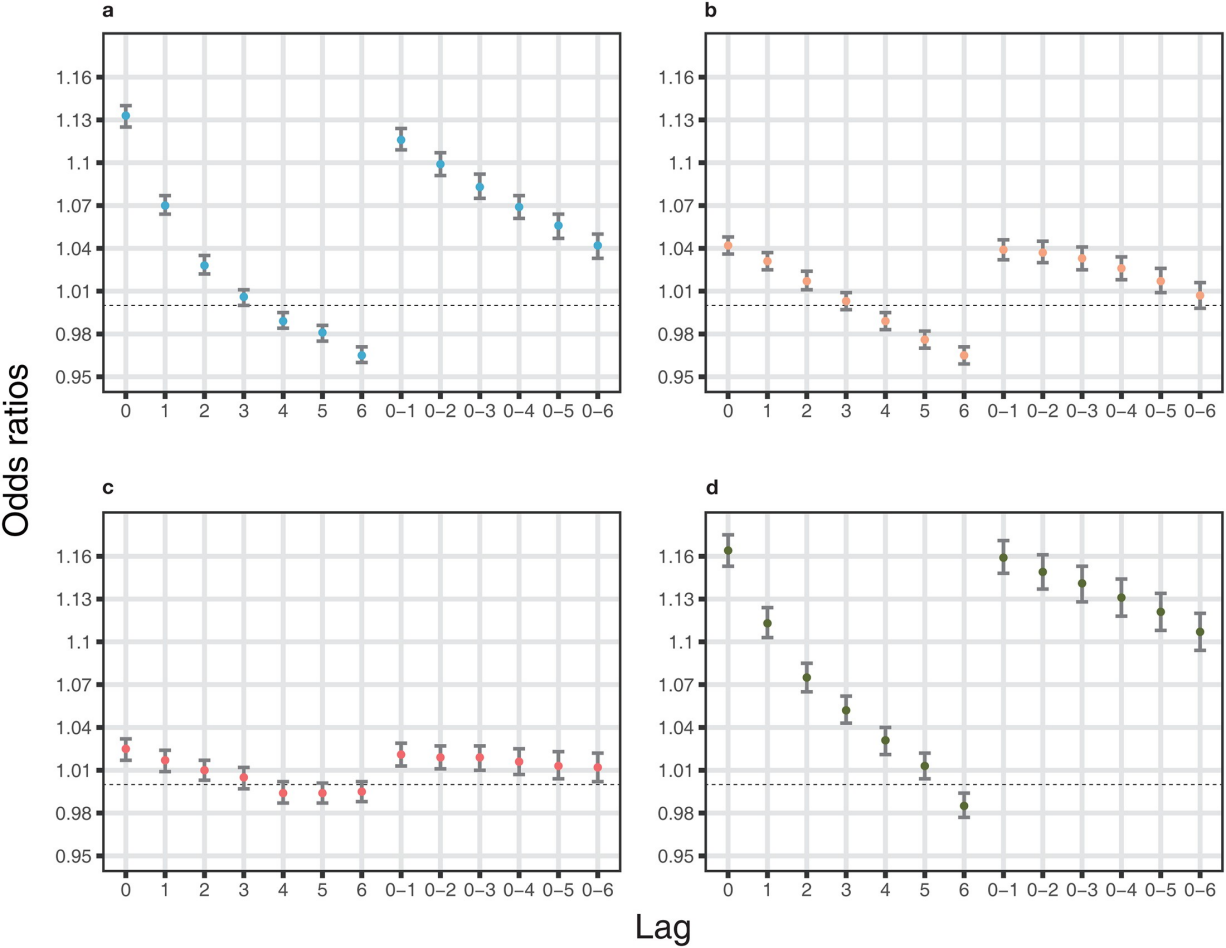
Risk of acute exacerbation of IBD due to ambient temperature change in NHIS cohort for each temperature quartile (a. cold quartile [-19.4°C–4.3°C], b. cool quartile [4.3–13.7], c. warm quartile [13.7–21.3], d. hot quartile [21.3–33.5]). The risk is described via ORs (dot) with 95% CIs (bar) through single lags (0 to 6) and moving averages (0–1 to 0–6). ORs are estimated with adjustment for daily relative humidity, PM_10_, NO_2_, SO_2_, CO and O_3_, per 1 °C decrease in daily average temperature for a; per 1 °C increase in daily average temperature for b, c and d. *Abbreviations: IBD, inflammatory bowel disease; NHIS, National Health Insurance Service; OR, odds ratio; CI, confidence interval.

**Fig 3:**
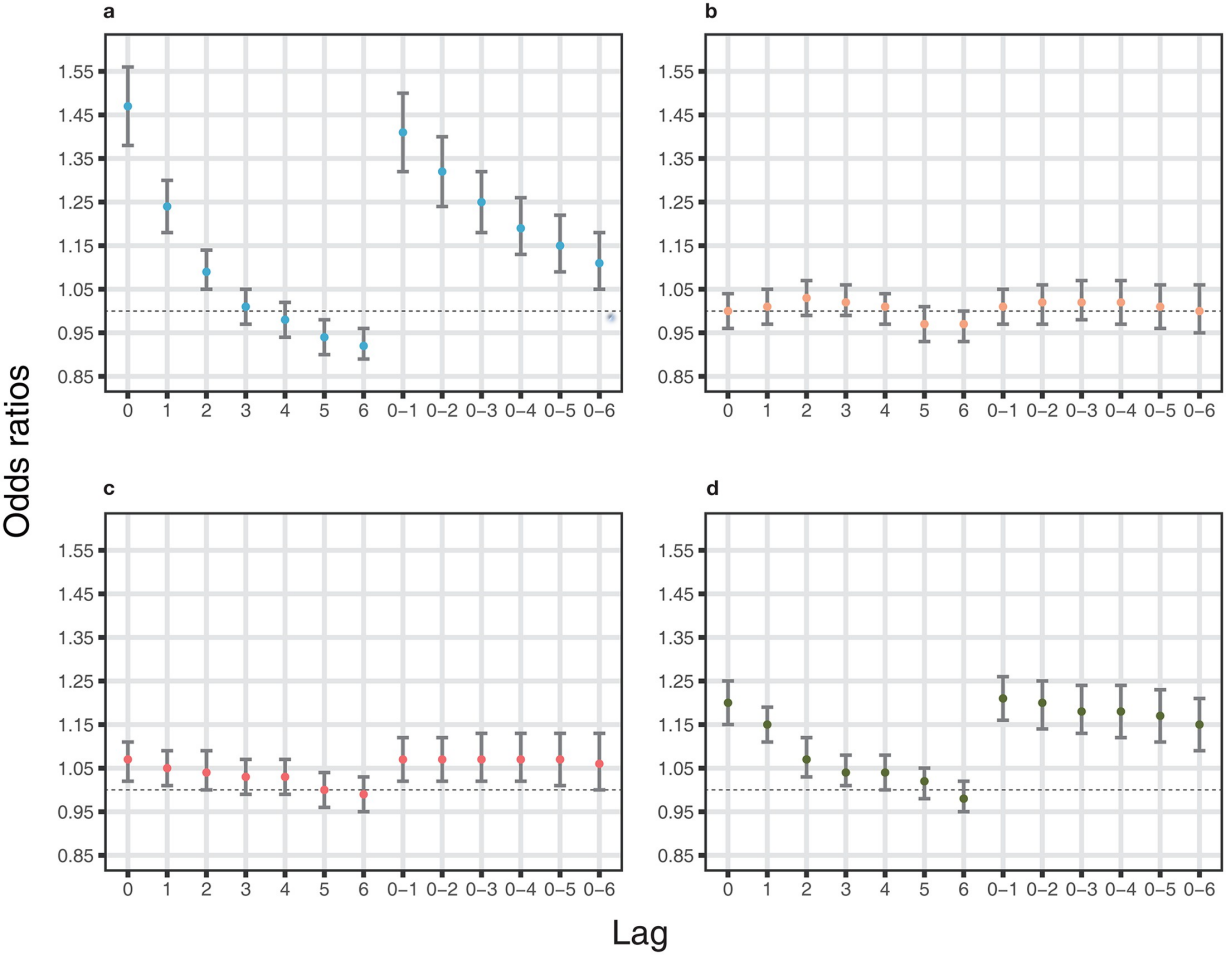
Risk of acute exacerbation of IBD due to ambient temperature change in UK Biobank cohort for each temperature quartile (a. cold quartile [-13.4°C–5.5°C], b. cool quartile [5.5–9.2], c. warm quartile [9.2–13.0], d. hot quartile [13.0–28.4]). The risk is described via ORs (dot) with 95% CIs (bar) through single lags (0 to 6) and moving averages (0–1 to 0–6). ORs are estimated with no adjustment per 1 °C decrease in daily average temperature for a; per 1 °C increase in daily average temperature for b, c and d. *Abbreviations: IBD, inflammatory bowel disease; OR, odds ratio; CI, confidence interval.

**Fig 4:**
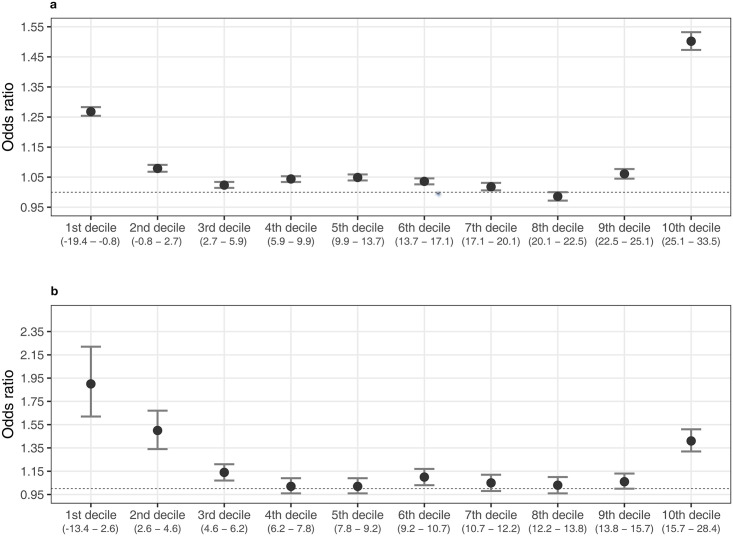
Risk of acute exacerbation of IBD due to ambient temperature change across temperature deciles in two large cohorts (a. NHIS cohort, b. UK Biobank cohort). The risk is described via ORs (dot) with 95% CIs (bar) when assuming no lag effect (lag 0). ORs are estimated with adjustment for daily relative humidity, PM_10_, NO_2_, SO_2_, CO and O_3_ in a; with no adjustment in b. The ambient temperature changes are considered per 1 °C decrease in daily average temperature for the first two deciles; per 1 °C increase in daily average temperature for the last eight deciles. *Abbreviations: IBD, inflammatory bowel disease; NHIS, National Health Insurance Service; OR, odds ratio; CI, confidence interval.

Acute exacerbation of IBD was more likely to be aggravated in the first and fourth quartiles of ambient temperature. In the first quartile, short-term temperature change was statistically associated with acute exacerbation of IBD in the NHIS database (Table 3; model 3: OR = 1.13, 95% confidence interval [CI]: 1.13–1.14 per 1°C decrease at single lag 0). Ambient temperature was statistically significant at shorter single lag times with a positive association, in contrast, at longer single lag times with a negative association. A statistically positive tendency for ORs was shown at moving averages in the NHIS database (Fig 2). In the UK Biobank, we also found a significant association between short-term temperature change and acute exacerbation of IBD in the crude model (Table 3; OR = 1.47, 95% CI: 1.38–1.56). In the fourth quartile, there was a significant positive association between the risk of acute exacerbation of IBD and short-term temperature change (Table 4; NHIS database: OR = 1.16, 95% CI: 1.15–1.17; UK Biobank: OR = 1.20, 95% CI: 1.15–1.25 per 1°C increase at single lag 0). ORs decreased with longer lags in the same pattern between the NHIS database and UK Biobank (NHIS database, model 3 at lag 6: OR = 0.98, 95% CI: 0.98–0.99; UK Biobank, crude model at lag 6: OR = 0.98, 95% CI: 0.95–1.02). Statistically positive associations were shown in all moving averages in both the NHIS database and UK Biobank (Fig 3). Interestingly, the ORs in the second and third quartiles of ambient temperature were relatively lower than those in the first and fourth quartiles (S2 and S3 Tables). We further examined the effect of short-term changes in ambient temperature on acute exacerbation of IBD across deciles of temperatures (Fig 4). The effect in the colder or warmer temperatures was higher than that in the moderate temperatures (S4 and S5 Tables).

**Table 3:** Odds ratios (95% CI) for acute exacerbation of IBD per 1 °C daily average temperature *decrease* at the first quartile.

**Dataset**	**Single–lag**	**Lag 0**	**Lag 1**	**Lag 2**	**Lag 3**	**Lag 4**	**Lag 5**	**Lag 6**
NHIS	Crude	1.12 (1.12–1.13)	1.08 (1.08–1.09)	1.04 (1.03–1.04)	1.01 (1.00–1.01)	0.99 (0.99–1.00)	0.99 (0.98–0.99)	0.97 (0.96–0.97)
NHIS	Model 1	1.12 (1.11–1.13)	1.08 (1.07–1.08)	1.04 (1.03–1.04)	1.01 (1.01–1.02)	1.00 (0.99–1.00)	0.99 (0.99–1.00)	0.97 (0.97–0.98)
NHIS	Model 2	1.12 (1.12–1.13)	1.07 (1.07–1.08)	1.04 (1.03–1.04)	1.01 (1.01–1.02)	0.99 (0.99–1.00)	0.99 (0.98–0.99)	0.97 (0.97–0.98)
NHIS	Model 3	1.13 (1.13–1.14)	1.07 (1.06–1.08)	1.03 (1.02–1.03)	1.01 (1.01–1.01)	0.99 (0.98–0.99)	0.98 (0.98–0.99)	0.97 (0.96–0.97)
UK Biobank	Crude	1.47 (1.38–1.56)	1.24 (1.18–1.30)	1.09 (1.05–1.14)	1.01 (0.97–1.05)	0.98 (0.94–1.02)	0.94 (0.90–0.98)	0.92 (0.89–0.96)
**Dataset**	**Moving average**		**Lag 0–1**	**Lag 0–2**	**Lag 0–3**	**Lag 0–4**	**Lag 0–5**	**Lag 0–6**
NHIS	Crude		1.12 (1.12–1.13)	1.12 (1.11–1.12)	1.10 (1.10–1.11)	1.09 (1.08–1.10)	1.08 (1.07–1.08)	1.06 (1.05–1.07)
NHIS	Model 1		1.11 (1.11–1.12)	1.11 (1.10–1.11)	1.09 (1.09–1.10)	1.08 (1.07–1.09)	1.07 (1.06–1.08)	1.06 (1.05–1.06)
NHIS	Model 2		1.12 (1.11–1.12)	1.11 (1.10–1.11)	1.09 (1.08–1.10)	1.08 (1.07–1.09)	1.07 (1.06–1.07)	1.05 (1.04–1.06)
NHIS	Model 3		1.12 (1.11–1.12)	1.10 (1.09–1.11)	1.08 (1.08–1.09)	1.07 (1.06–1.08)	1.06 (1.05–1.06)	1.04 (1.03–1.05)
UK Biobank	Crude		1.41 (1.32–1.50)	1.32 (1.24–1.40)	1.25 (1.18–1.32)	1.19 (1.13–1.26)	1.15 (1.09–1.22)	1.11 (1.05–1.18)

* Model 1: with adjustment for relative humidity.

Model 2: Model 1 with further adjustment for PM_10_.

Model 3: Model 2 with further adjustment for NO_2_, SO_2_, O_3_ and CO.

**Table 4:** Odds ratios (95% CI) for acute exacerbation of IBD per 1 °C daily average temperature increase at the fourth quartile.

**Dataset**	**Single–lag**	**Lag 0**	**Lag 1**	**Lag 2**	**Lag 3**	**Lag 4**	**Lag 5**	**Lag 6**
NHIS	Crude	1.16 (1.15–1.17)	1.10 (1.09–1.11)	1.06 (1.05–1.07)	1.04 (1.03–1.05)	1.02 (1.01–1.03)	1.00 (1.00–1.01)	0.98 (0.97–0.99)
NHIS	Model 1	1.16 (1.15–1.17)	1.11 (1.10–1.12)	1.07 (1.06–1.08)	1.05 (1.04–1.06)	1.03 (1.02–1.04)	1.01 (1.00–1.02)	0.98 (0.97–0.99)
NHIS	Model 2	1.16 (1.15–1.17)	1.11 (1.10–1.12)	1.07 (1.06–1.08)	1.05 (1.04–1.06)	1.03 (1.02–1.04)	1.01 (1.00–1.02)	0.98 (0.98–0.99)
NHIS	Model 3	1.16 (1.15–1.17)	1.11 (1.10–1.12)	1.08 (1.07–1.08)	1.05 (1.04–1.06)	1.03 (1.02–1.04)	1.01 (1.00–1.02)	0.98 (0.98–0.99)
UK Biobank	Crude	1.20 (1.15–1.25)	1.15 (1.11–1.19)	1.07 (1.03–1.12)	1.04 (1.01–1.08)	1.04 (1.00–1.08)	1.02 (0.98–1.05)	0.98 (0.95–1.02)
**Dataset**	**Moving average**		**Lag 0–1**	**Lag 0–2**	**Lag 0–3**	**Lag 0–4**	**Lag 0–5**	**Lag 0–6**
NHIS	Crude		1.16 (1.15–1.17)	1.14 (1.13–1.15)	1.13 (1.12–1.15)	1.12 (1.11–1.14)	1.11 (1.10–1.12)	1.10 (1.09–1.11)
NHIS	Model 1		1.16 (1.15–1.17)	1.15 (1.13–1.16)	1.14 (1.12–1.15)	1.13 (1.11–1.14)	1.11 (1.10–1.13)	1.10 (1.09–1.11)
NHIS	Model 2		1.16 (1.15–1.17)	1.14 (1.13–1.16)	1.14 (1.12–1.15)	1.13 (1.11–1.14)	1.11 (1.10–1.13)	1.10 (1.09–1.11)
NHIS	Model 3		1.16 (1.15–1.17)	1.15 (1.14–1.16)	1.14 (1.13–1.15)	1.13 (1.12–1.14)	1.12 (1.11–1.13)	1.11 (1.09–1.12)
UK Biobank	Crude		1.21 (1.16–1.26)	1.20 (1.14–1.25)	1.18 (1.13–1.24)	1.18 (1.12 1.24)	1.17 (1.11–1.23)	1.15 (1.09–1.21)

* Model 1: with adjustment for relative humidity.

Model 2: Model 1 with further adjustment for PM_10_.

Model 3: Model 2 with further adjustment for NO_2_, SO_2_, O_3_ and CO.

The sensitivity analyses for CD or UC patient groups showed similar results to those for the entire patients in the NHIS database (S6 and S7 Tables). The risk of acute exacerbation due to the ambient temperature in the first and fourth quartiles was typically higher than that second and third in quartiles. However, when the lag time was longer, the ORs decreased in every quartile.

## Discussion

We revealed that the short-term change toward severe temperatures was more likely to aggravate acute exacerbation of IBD, specifically in the cold (the first quartile of ambient temperature) and hot weathers (the fourth quartile). The effect on acute exacerbation of IBD decreased significantly from lag 0 to lag 6. The risk trends were more evident than those in the cool and warm weathers while they were consistent in the subgroup analyses of patients with CD and UC. These results demonstrate that the acute exacerbation of IBD had a seasonal pattern, and was affected by the short-term temperature change in cold and hot weathers rather than mild weathers.

Available evidence suggests that environmental factors have variable effects on the risk of IBD and/or the risk of disease progression [16]. Environmental factors are the possible cause for the increased incidence of IBD in recent years, especially in East Asia, where the incidence of IBD was previously low. Various environmental factors, including climate and air pollution, have been identified as risk or protective factors for IBD [5, 16]. A previous study suggested that air temperature can change the microbial population of the human gut microbiome, increase the likelihood of an imbalance in the gut microbiome, and increase the likelihood of developing IBD [17].

With the recent increase in climate change awareness, there is a growing interest in environmental factors, especially the potential mechanisms by which temperature affects health [18–22]. The change in ambient temperature is deeply related to body temperature regulation, and even extreme heat can cause thermoregulatory mechanisms to fail, resulting in hyperthermia [23]. Conversely, thermoregulation breakdown in extreme cold temperatures leads to heat-related illnesses such as hypothermia. Moreover, gastrointestinal health is influenced by multiple issues related to climate change [24, 25]. In this study, we focused on the association between short-term changes in ambient temperature and acute exacerbation of IBD.

An increase of the physiological temperature shifts the gene expression response to tumor necrosis factor-α (TNF-α) and then frequent nuclear factor kappa-light-chain-enhancer of activated B cell (NF-κB) dynamics arise [26]. The dynamics of NF-κB oscillations related with cytokine stimulation can be modified by small temperature changes. These changes can also regulate the inflammatory response via NF-κB functions. Activated NF-κB proteins induce aggravation of intestinal inflammation [27]. Furthermore, the number of cells with activated NF-κB significantly correlates with the severity of mucosal inflammation [27, 28]. Moreover, body temperature changes affect regulation of the immune response [29, 30]. The immunological imbalance is related to IBD through TNF-α [31]. However, the relationship among thermogenesis, inflammatory response, and IBD aggravation has not been clarified. Therefore, we hypothesized that IBD may worsen by thermal stimuli through biological and immunological pathways.

The strengths of our study are as follows. First, we used a nationwide population-based cohort in South Korea and a prospective cohort in the UK. We believe that they were the largest cohorts used to investigate the association between acute exacerbation of IBD and ambient temperature, and estimate the seasonal variation in acute exacerbation of IBD. Second, we used a bidirectional case-crossover design to minimize implicit bias from confounding due to both unmeasured covariates between individuals and time trend. In the cohort from South Korea, we further adjusted other environmental factors, such as relative humidity and air pollutants. Third, we represented seasonality as quartiles of the empirical distribution for ambient temperature to investigate seasonal difference in the pattern of association between acute exacerbation of IBD and ambient temperature change. The directions of the association were reverse in cold vs hot weathers while no significant association was found in cool and warm weathers. Previous works were similar with U-shaped risk of ambient temperature for mortality or other gastrointestinal disease [22, 32, 33]. The opposite associations attenuate the risk of short-term temperature change, and result in no significant association overall when a whole range of temperatures were considered. Some studies on the association between seasonality and onset/acute exacerbation of IBD defined seasons by months [7, 34, 35]. However, seasonality by month differs according to geographical location.

There are some limitations in our study. First, the ambient temperature does not represent the individual body temperature. While body temperature is directly influenced by ambient temperature, it differs among individuals depending on the physiological or exposed environmental factors. Therefore, we used a bidirectional case-crossover design to control for bias due to this individual difference. Second, selection bias may have occurred owing to the operational definition of acute exacerbation of IBD. Because we used claim data rather than detailed medical records, the acute exacerbation of IBD was operationally defined. Thus, the study population may be under-/over-represented. Third, the association between ambient temperature and acute exacerbation of IBD can be observed due to unmeasured modifications of nutritional habits by changes of temperature. The aspect of comorbidities for the cohorts is different because the cohort from the NHIS is real-world data retrospectively collected from claims database, in contrast, the external data from the UK Biobank is a prospective cohort comprising generally healthy participants. However, the bias can be reduced by self-controlled design with short-term periods. Fourth, we assumed the day of the occurrence of the acute IBD exacerbation is the same as the day of the visit to the hospital and the diagnosis of the disease. However, it still has a limitation for the consensus for the day between the occurrence of the disease and the visit to the hospital. Therefore, we considered the induction period of zero to 6 lagged days with showing consistent results. While South Korea’s healthcare environment features relatively short waiting times for medical appointments, the UK may differ in this aspect. However, we applied the same study design to both countries due to the difficulty with measuring the induction period from the disease occurrence to the hospital-visit and our intention to minimize the effects of confounding. Fifth, the definition of acute IBD exacerbation might be limited because the disease was not designated with ICD-10 codes. Furthermore, we differently defined the disease due to the unavailability of prescription data in the UK Biobank.

## Conclusions

We investigated the association between ambient temperature and acute exacerbation of IBD using real-world claims data from South Korea and a prospective cohort from the UK. Our results provide evidence for the association in cold and hot weathers. However, epidemiological evidence is still lack of between the ambient temperature and the acute exacerbation of IBD. It needs to be revealed in other large cohorts. Meanwhile, future studies should explore the biological causation of body temperature changes due to ambient temperature changes leading to acute exacerbation of IBD.

## Supporting information

S1 Table ICD-10 codes for comorbidities. (DOCX)

S2 Table Odds ratios (95% CI) for acute exacerbation of IBD per 1 °C daily average temperature increase at the second quartile. (DOCX)

S3 Table Odds ratios (95% CI) for acute exacerbation of IBD per 1 °C daily average temperature increase at the third quartile. CI, confidence interval; IBD, inflammatory bowel disease.
(DOCX)

S4 Table Odds ratios (95% CI) for acute exacerbation of IBD per 1 °C daily average temperature change at the decile (°C) with NHIS. (DOCX)

S5 Table Odds ratios (95% CI) for acute exacerbation of IBD per 1 °C daily average temperature change at the decile (°C) with the UK Biobank. (DOCX)

S6 Table Odds ratios (95% CI) for acute exacerbation of Crohn’s disease per 1 °C daily average temperature change derived from Model 3. (DOCX)

S7 Table Odds ratios (95% CI) for acute exacerbation of ulcerative colitis per 1 °C daily average temperature change derived from Model 3. (DOCX)

S1 Checklist STROBE statement—Checklist of items that should be included in reports of *case-control studies*. (DOCX)
