# Supplementary material for: Short-term effects of ambient temperature on acute exacerbation of inflammatory bowel disease: A nationwide case-crossover study with external validation
Source: PLoS One. 2023 Dec 29;18(12):e0291713. doi: 10.1371/journal.pone.0291713 (PMC10756522; doi:10.1371/journal.pone.0291713)
Supplement: S1 Table — (DOCX) [file pone.0291713.s001.docx]

**S1 Table.** ICD-10 codes for comorbidities.

| **Covariates** | **ICD-10 code** |
| --- | --- |
| Myocardial infarction | I21, I22, I252 |
| Congestive heart failure | I43, I50, I099, I110, I130, I132, I255, I420, I425, I426, I427, I428, I429, P290 |
| Peripheral vascular dis. | I70, I71, I731, I738, I739, I771, I790, I792, K551, K558, K559, Z958, Z959 |
| Cerebrovascular dis. | G45, G46, I60, I61, I62, I63, I64, I65, I66, I67, I68, I69, H340 |
| Dementia | F00, F01, F02, F03, G30, F051, G311 |
| Chronic pulmonary dis. | J40, J41, J42, J43, J44, J45, J46, J47, J60, J61, J62, J63, J64, J65, J66, J67, I278, I279, J684, J701, J703 |
| Connective tissue dis. | M05, M32, M33, M34, M06, M315, M351, M353, M360 |
| Peptic ulcer dis. | K25, K26, K27, K28 |
| Diabetes (without complications) | E100, E101, E106, E108, E109, E110, E111, E116, E118, E119, E120, E121, E126, E128, E129, E130, E131, E136, E138, E139, E140, E141, E146, E148, E149 |
| Diabetes (with complications) | E102, E103, E104, E105, E107, E112, E113, E114, E115, E117, E122, E123, E124, E125, E127, E132, E133, E134, E135, E137, E142, E143, E144, E145, E147 |
| Paraplegia/hemiplegia | G81, G82, G041, G114, G800, G830, G831, G832, G833, G834, G839 |
| Mild liver disease | B18, K73, K74, K700, K701, K702, K703, K709, K717, K713, K714, K715, K760, K762, K763, K764, K768, K769, Z944 |
| Moderate or severe liver disease | K704, K711, K721, K729, K765, K766, K767, I850, I859, I864, I982 |
| Renal disease | N18, N19, N052, N053, N054, N055, N056, N057, N250, I120, I131, N032, N033, N034, N035, N036, N037, Z490, Z491, Z492, Z940, Z992 |
| Malignancy | All C codes |
| AIDS | B20, B21, B22, B24 |

ICD-10, International Classification of Diseases, 10th Revision; AIDS, acquired immune deficiency syndrome.
