# Supplementary material for: Short-term effects of ambient temperature on acute exacerbation of inflammatory bowel disease: A nationwide case-crossover study with external validation
Source: PLoS One. 2023 Dec 29;18(12):e0291713. doi: 10.1371/journal.pone.0291713 (PMC10756522; doi:10.1371/journal.pone.0291713)
Supplement: S2 Table — (DOCX) [file pone.0291713.s002.docx]

**S2 Table.** Odds ratios (95% CI) for acute exacerbation of IBD per 1 ºC daily average temperature increase at the second quartile.

| **Dataset** | **Single-lag** | **Lag 0** | **Lag 1** | **Lag 2** | **Lag 3** | **Lag 4** | **Lag 5** | **Lag 6** |
| --- | --- | --- | --- | --- | --- | --- | --- | --- |
| NHIS | Crude | 1.05 (1.05–1.06) | 1.04 (1.04–1.05) | 1.02 (1.02–1.03) | 1.01 (1.00–1.01) | 1.00 (0.99–1.00) | 0.98 (0.98–0.99) | 0.97 (0.96–0.97) |
| NHIS | Model 1 | 1.04 (1.04–1.05) | 1.03 (1.03–1.04) | 1.02 (1.01–1.02) | 1.01 (1.00–1.01) | 1.00 (0.99–1.00) | 0.98 (0.98–0.99) | 0.97 (0.96–0.97) |
| NHIS | Model 2 | 1.04 (1.03–1.05) | 1.03 (1.03–1.04) | 1.02 (1.01–1.02) | 1.01 (1.00–1.01) | 0.99 (0.99–1.00) | 0.98 (0.98–0.99) | 0.97 (0.97–0.98) |
| NHIS | Model 3 | 1.04 (1.04–1.05) | 1.03 (1.02–1.04) | 1.02 (1.01–1.02) | 1.00 (1.00–1.01) | 0.99 (0.98–1.00) | 0.98 (0.97–0.98) | 0.96 (0.96–0.97) |
| UK Biobank | Crude | 1.00 (0.96–1.04) | 1.01 (0.97–1.05) | 1.03 (0.99–1.07) | 1.02 (0.99–1.06) | 1.01 (0.97–1.04) | 0.97 (0.93–1.01) | 0.97 (0.93–1.00) |
| **Dataset** | **Moving average** |  | **Lag 0–1** | **Lag 0–2** | **Lag 0–3** | **Lag 0–4** | **Lag 0–5** | **Lag 0–6** |
| NHIS | Crude |  | 1.06 (1.05–1.06) | 1.05 (1.05–1.06) | 1.05 (1.04–1.05) | 1.04 (1.03–1.05) | 1.03 (1.02–1.04) | 1.02 (1.01–1.03) |
| NHIS | Model 1 |  | 1.04 (1.03–1.05) | 1.04 (1.03–1.04) | 1.03 (1.03–1.04) | 1.03 (1.02–1.03) | 1.02 (1.01–1.03) | 1.01 (1.00–1.02) |
| NHIS | Model 2 |  | 1.04 (1.03–1.05) | 1.04 (1.03–1.04) | 1.03 (1.02–1.04) | 1.02 (1.02–1.03) | 1.02 (1.01–1.02) | 1.01 (1.00–1.01) |
| NHIS | Model 3 |  | 1.04 (1.03–1.05) | 1.04 (1.03–1.04) | 1.03 (1.02–1.04) | 1.03 (1.02–1.03) | 1.02 (1.01–1.03) | 1.01 (1.00–1.02) |
| UK Biobank | Crude |  | 1.01 (0.97–1.05) | 1.02 (0.97–1.06) | 1.02 (0.98–1.07) | 1.02 (0.97–1.07) | 1.01 (0.96–1.06) | 1.00 (0.95–1.06) |

* Model 1: with adjustment for relative humidity.
Model 2: Model 1 with further adjustment for PM_10_.
Model 3: Model 2 with further adjustment for NO_2_, SO_2_, O_3_ and CO.
