# Supplementary material for: Short-term effects of ambient temperature on acute exacerbation of inflammatory bowel disease: A nationwide case-crossover study with external validation
Source: PLoS One. 2023 Dec 29;18(12):e0291713. doi: 10.1371/journal.pone.0291713 (PMC10756522; doi:10.1371/journal.pone.0291713)
Supplement: S4 Table — (DOCX) [file pone.0291713.s004.docx]

**S4 Table.** Odds ratios (95% CI) for acute exacerbation of IBD per 1 ºC daily average temperature change at the decile (ºC) with NHIS.

| **Single-lag** | **Lag 0** | **Lag 1** | **Lag 2** | **Lag 3** | **Lag 4** | **Lag 5** | **Lag 6** |
| --- | --- | --- | --- | --- | --- | --- | --- |
| 1st decile (-19.4 – -0.8) | 1.27 (1.25–1.28) | 1.12 (1.11–1.14) | 1.04 (1.03–1.05) | 1.01 (1.00–1.01) | 0.98 (0.97–0.99) | 0.96 (0.95–0.96) | 0.94 (0.93–0.94) |
| 2nd decile (-0.8 – 2.7) | 1.08 (1.07–1.09) | 1.04 (1.03–1.05) | 1.01 (1.00–1.02) | 1.00 (0.99–1.01) | 0.99 (0.98–1.00) | 1.00 (0.99–1.01) | 0.98 (0.97–0.99) |
| 3rd decile (2.7 – 5.9) | 1.02 (1.01–1.03) | 1.00 (0.99–1.01) | 0.99 (0.98–1.00) | 0.99 (0.98–1.00) | 0.99 (0.98–1.00) | 1.00 (0.99–1.01) | 0.99 (0.98–1.00) |
| 4th decile (5.9 – 9.9) | 1.04 (1.03–1.05) | 1.04 (1.03–1.05) | 1.03 (1.02–1.04) | 1.01 (1.00–1.02) | 0.99 (0.98–1.00) | 0.97 (0.96–0.98) | 0.96 (0.95–0.97) |
| 5th decile (9.9 – 13.7) | 1.05 (1.04–1.06) | 1.04 (1.03–1.05) | 1.02 (1.01–1.03) | 1.00 (0.99–1.01) | 0.98 (0.97–0.99) | 0.96 (0.96–0.97) | 0.96 (0.95–0.97) |
| 6th decile (13.7 – 17.1) | 1.04 (1.03–1.05) | 1.02 (1.01–1.03) | 1.01 (1.00–1.02) | 0.99 (0.98–1.00) | 0.98 (0.98–1.00) | 0.99 (0.98–1.00) | 0.99 (0.98–1.00) |
| 7th decile (17.1 – 20.1) | 1.02 (1.01–1.03) | 1.01 (1.00–1.02) | 1.01 (1.00–1.02) | 1.02 (1.01–1.03) | 1.01 (1.00–1.02) | 1.00 (0.99–1.02) | 1.01 (1.00–1.02) |
| 8th decile (20.1 – 22.5) | 0.99 (0.97–1.00) | 0.98 (0.97–1.00) | 0.99 (0.97–1.00) | 1.00 (0.98–1.01) | 0.99 (0.97–1.00) | 0.98 (0.97–1.00) | 0.98 (0.96–0.99) |
| 9th decile (22.5 – 25.1) | 1.06 (1.04–1.08) | 1.04 (1.02–1.05) | 1.02 (1.00–1.03) | 1.01 (0.99–1.02) | 1.01 (0.99–1.02) | 0.99 (0.98–1.01) | 0.99 (0.98–1.00) |
| 10th decile (25.1 – 33.5) | 1.5 (1.47–1.53) | 1.34 (1.32–1.37) | 1.23 (1.21–1.25) | 1.16 (1.14–1.18) | 1.09 (1.07–1.11) | 1.05 (1.04–1.07) | 0.98 (0.96–1.00) |
| **Moving average** |  | **Lag 0–1** | **Lag 0–2** | **Lag 0–3** | **Lag 0–4** | **Lag 0–5** | **Lag 0–6** |
| 1st decile (-19.4 – -0.8) |  | 1.22 (1.21–1.24) | 1.18 (1.16–1.19) | 1.14 (1.13–1.16) | 1.12 (1.11–1.13) | 1.09 (1.08–1.11) | 1.07 (1.05–1.08) |
| 2nd decile (-0.8 – 2.7) |  | 1.07 (1.05–1.08) | 1.05 (1.04–1.07) | 1.04 (1.03–1.06) | 1.03 (1.02–1.04) | 1.03 (1.01–1.04) | 1.02 (1.01–1.03) |
| 3rd decile (2.7 – 5.9) |  | 1.02 (1.01–1.03) | 1.02 (1.01–1.03) | 1.01 (1.00–1.02) | 1.01 (1.00–1.02) | 1.01 (0.99–1.02) | 1.01 (1.00–1.02) |
| 4th decile (5.9 – 9.9) |  | 1.05 (1.04–1.06) | 1.05 (1.04–1.06) | 1.05 (1.03–1.06) | 1.04 (1.03–1.05) | 1.03 (1.01–1.04) | 1.02 (1.00–1.03) |
| 5th decile (9.9 – 13.7) |  | 1.04 (1.03–1.05) | 1.04 (1.02–1.05) | 1.03 (1.02–1.04) | 1.02 (1.01–1.03) | 1.01 (0.99–1.02) | 1.00 (0.98–1.01) |
| 6th decile (13.7 – 17.1) |  | 1.03 (1.02–1.04) | 1.03 (1.02–1.04) | 1.02 (1.01–1.04) | 1.02 (1.00–1.03) | 1.01 (1.00–1.02) | 1.01 (0.99–1.02) |
| 7th decile (17.1 – 20.1) |  | 1.01 (1.00–1.03) | 1.01 (1.00–1.03) | 1.02 (1.00–1.03) | 1.02 (1.01–1.04) | 1.02 (1.01–1.04) | 1.03 (1.01–1.04) |
| 8th decile (20.1 – 22.5) |  | 0.98 (0.97–1.00) | 0.98 (0.97–1.00) | 0.99 (0.97–1.00) | 0.98 (0.97–1.00) | 0.98 (0.97–1.00) | 0.98 (0.96–1.00) |
| 9th decile (22.5 – 25.1) |  | 1.06 (1.04–1.07) | 1.05 (1.03–1.07) | 1.04 (1.02–1.06) | 1.04 (1.02–1.06) | 1.03 (1.01–1.05) | 1.03 (1.01–1.05) |
| 10th decile (25.1 – 33.5) |  | 1.49 (1.46–1.52) | 1.45 (1.42–1.48) | 1.42 (1.39–1.45) | 1.39 (1.36–1.42) | 1.36 (1.33–1.39) | 1.32 (1.29–1.35) |
