# Supplementary material for: Short-term effects of ambient temperature on acute exacerbation of inflammatory bowel disease: A nationwide case-crossover study with external validation
Source: PLoS One. 2023 Dec 29;18(12):e0291713. doi: 10.1371/journal.pone.0291713 (PMC10756522; doi:10.1371/journal.pone.0291713)
Supplement: S5 Table — (DOCX) [file pone.0291713.s005.docx]

**S5 Table.** Odds ratios (95% CI) for acute exacerbation of IBD per 1 ºC daily average temperature change at the decile (ºC) with the UK Biobank.

| **Single-lag** | **Lag 0** | **Lag 1** | **Lag 2** | **Lag 3** | **Lag 4** | **Lag 5** | **Lag 6** |
| --- | --- | --- | --- | --- | --- | --- | --- |
| 1st decile (-13.4 – 2.6) | 1.90 (1.62–2.22) | 1.42 (1.29–1.58) | 1.20 (1.11–1.30) | 1.10 (1.02–1.18) | 1.03 (0.96–1.10) | 0.94 (0.88–1.01) | 0.90 (0.85–0.97) |
| 2nd decile (2.6 – 4.6) | 1.50 (1.34–1.67) | 1.20 (1.12–1.30) | 1.04 (0.97–1.11) | 0.94 (0.88–1.00) | 0.93 (0.87–0.99) | 0.90 (0.84–0.97) | 0.88 (0.82–0.95) |
| 3rd decile (4.6 – 6.2) | 1.14 (1.07–1.21) | 1.07 (1.01–1.14) | 1.03 (0.97–1.09) | 1.02 (0.96–1.07) | 1.01 (0.96–1.06) | 1.03 (0.97–1.09) | 1.04 (0.98–1.11) |
| 4th decile (6.2 – 7.8) | 1.02 (0.96–1.09) | 1.05 (0.99–1.12) | 1.05 (0.99–1.12) | 1.04 (0.98–1.10) | 1.00 (0.94–1.05) | 0.97 (0.91–1.02) | 0.94 (0.88–1.00) |
| 5th decile (7.8 – 9.2) | 1.02 (0.96–1.09) | 1.01 (0.94–1.07) | 1.02 (0.95–1.09) | 1.05 (0.98–1.12) | 1.05 (0.98–1.12) | 0.99 (0.93–1.06) | 1.01 (0.95–1.07) |
| 6th decile (9.2 – 10.7) | 1.10 (1.03–1.17) | 1.10 (1.03–1.17) | 1.06 (1.00–1.13) | 1.03 (0.97–1.00) | 1.02 (0.96–1.08) | 0.99 (0.93–1.05) | 0.99 (0.93–1.05) |
| 7th decile (10.7 – 12.2) | 1.05 (0.98–1.12) | 1.00 (0.94–1.06) | 1.02 (0.95–1.09) | 1.03 (0.96–1.11) | 1.03 (0.96–1.10) | 1.00 (0.94–1.07) | 1.02 (0.95–1.09) |
| 8th decile (12.2 – 13.8) | 1.03 (0.96–1.10) | 1.06 (0.99–1.14) | 1.03 (0.96–1.10) | 1.00 (0.94–1.07) | 1.04 (0.97–1.10) | 1.00 (0.94–1.06) | 0.95 (0.89–1.01) |
| 9th decile (13.8 – 15.7) | 1.06 (1.00–1.13) | 1.07 (1.01–1.14) | 1.01 (0.95–1.08) | 1.00 (0.94–1.06) | 1.00 (0.94–1.06) | 1.03 (0.97–1.09) | 1.00 (0.94–1.06) |
| 10th decile (15.7 – 28.4) | 1.41 (1.32–1.51) | 1.25 (1.18–1.33) | 1.15 (1.09–1.22) | 1.10 (1.05–1.17) | 1.07 (1.02–1.13) | 1.02 (0.96–1.07) | 0.98 (0.93–1.03) |
| **Moving average** |  | **Lag 0–1** | **Lag 0–2** | **Lag 0–3** | **Lag 0–4** | **Lag 0–5** | **Lag 0–6** |
| 1st decile (-13.4 – 2.6) |  | 1.76 (1.53–2.04) | 1.61 (1.41–1.83) | 1.49 (1.32–1.67) | 1.40 (1.25–1.57) | 1.31 (1.18–1.45) | 1.23 (1.11–1.36) |
| 2nd decile (2.6 – 4.6) |  | 1.40 (1.27–1.55) | 1.27 (1.16–1.40) | 1.18 (1.08–1.29) | 1.12 (1.02–1.23) | 1.07 (0.98–1.18) | 1.03 (0.94–1.14) |
| 3rd decile (4.6 – 6.2) |  | 1.12 (1.05–1.20) | 1.10 (1.03–1.18) | 1.09 (1.01–1.17) | 1.07 (1.00–1.15) | 1.07 (1.00–1.16) | 1.08 (1.00–1.17) |
| 4th decile (6.2 – 7.8) |  | 1.04 (0.98–1.12) | 1.06 (0.99–1.14) | 1.07 (0.99–1.15) | 1.05 (0.98–1.14) | 1.04 (0.96–1.12) | 1.02 (0.94–1.10) |
| 5th decile (7.8 – 9.2) |  | 1.02 (0.95–1.09) | 1.02 (0.95–1.10) | 1.04 (0.96–1.12) | 1.05 (0.96–1.15) | 1.05 (0.95–1.15) | 1.05 (0.95–1.15) |
| 6th decile (9.2 – 10.7) |  | 1.12 (1.04–1.19) | 1.12 (1.04–1.20) | 1.11 (1.03–1.20) | 1.10 (1.02–1.20) | 1.09 (1.00–1.19) | 1.08 (0.99–1.19) |
| 7th decile (10.7 – 12.2) |  | 1.02 (0.95–1.10) | 1.03 (0.95–1.10) | 1.03 (0.95–1.12) | 1.04 (0.95–1.13) | 1.04 (0.95–1.14) | 1.04 (0.95–1.15) |
| 8th decile (12.2 – 13.8) |  | 1.05 (0.98–1.14) | 1.05 (0.97–1.14) | 1.05 (0.96–1.14) | 1.06 (0.97–1.16) | 1.05 (0.96–1.15) | 1.03 (0.93–1.14) |
| 9th decile (13.8 – 15.7) |  | 1.08 (1.01–1.16) | 1.07 (0.99–1.14) | 1.05 (0.98–1.14) | 1.05 (0.97–1.14) | 1.05 (0.97–1.15) | 1.05 (0.96–1.15) |
| 10th decile (15.7 – 28.4) |  | 1.40 (1.30–1.50) | 1.37 (1.28–1.48) | 1.36 (1.26–1.47) | 1.34 (1.24–1.45) | 1.32 (1.22–1.43) | 1.28 (1.18–1.39) |
