# Supplementary material for: Short-term effects of ambient temperature on acute exacerbation of inflammatory bowel disease: A nationwide case-crossover study with external validation
Source: PLoS One. 2023 Dec 29;18(12):e0291713. doi: 10.1371/journal.pone.0291713 (PMC10756522; doi:10.1371/journal.pone.0291713)
Supplement: S7 Table — (DOCX) [file pone.0291713.s007.docx]

**S7 Table.** Odds ratios (95% CI) for acute exacerbation of ulcerative colitis per 1 ºC daily average temperature change derived from Model 3.

| **Single-lag** | **Lag 0** | **Lag 1** | **Lag 2** | **Lag 3** | **Lag 4** | **Lag 5** | **Lag 6** |
| --- | --- | --- | --- | --- | --- | --- | --- |
| Q1 (-19.4 – 4.3) | 1.13 (1.12–1.14) | 1.07 (1.06–1.08) | 1.03 (1.02–1.04) | 1.00 (1.00–1.01) | 0.99 (0.98–1.00) | 0.98 (0.98–0.99) | 0.97 (0.96–0.98) |
| Q2 (4.3 – 13.7) | 1.04 (1.03–1.05) | 1.03 (1.02–1.04) | 1.02 (1.01–1.02) | 1.00 (0.99–1.01) | 0.99 (0.98–1.00) | 0.98 (0.97–0.98) | 0.97 (0.96–0.97) |
| Q3 (13.7 – 21.3) | 1.02 (1.01–1.03) | 1.02 (1.01–1.03) | 1.01 (1.00–1.02) | 1.01 (1.00–1.01) | 0.99 (0.99–1.00) | 0.99 (0.98–1.00) | 0.99 (0.98–1.00) |
| Q4 (21.3 – 33.5) | 1.16 (1.15–1.18) | 1.11 (1.1–1.12) | 1.07 (1.06–1.08) | 1.05 (1.03–1.06) | 1.03 (1.01–1.04) | 1.01 (1.00–1.02) | 0.98 (0.97–1.00) |
| **Moving average** |  | **Lag 0–1** | **Lag 0–2** | **Lag 0–3** | **Lag 0–4** | **Lag 0–5** | **Lag 0–6** |
| Q1 (-19.4 – 4.3) |  | 1.11 (1.10–1.12) | 1.10 (1.09–1.11) | 1.08 (1.07–1.09) | 1.07 (1.06–1.08) | 1.06 (1.05–1.07) | 1.04 (1.03–1.05) |
| Q2 (4.3 – 13.7) |  | 1.04 (1.03–1.05) | 1.04 (1.03–1.05) | 1.03 (1.02–1.04) | 1.03 (1.02–1.04) | 1.02 (1.01–1.03) | 1.01 (1.00–1.02) |
| Q3 (13.7 – 21.3) |  | 1.02 (1.01–1.03) | 1.02 (1.01–1.03) | 1.02 (1.01–1.03) | 1.02 (1.00–1.03) | 1.01 (1.00–1.02) | 1.01 (1.00–1.02) |
| Q4 (21.3 – 33.5) |  | 1.16 (1.14–1.17) | 1.15 (1.13–1.16) | 1.14 (1.12–1.15) | 1.12 (1.11–1.14) | 1.11 (1.10–1.13) | - 1. (1.08–1.12) |

Model 3: Daily average temperature + daily relative humidity + PM10 + NO2 + SO2 + O3 + CO
